# Exploring Coronary Artery Disease GWAs Targets With Functional Links to Immunometabolism

**DOI:** 10.3389/fcvm.2018.00148

**Published:** 2018-11-06

**Authors:** Maria F. Hughes, Yvonne M. Lenighan, Catherine Godson, Helen M. Roche

**Affiliations:** ^1^UCD Diabetes Complications Research Centre, Conway Institute of Biomolecular and Biomedical Research, University College Dublin, Dublin, Ireland; ^2^Nutrigenomics Research Group, UCD Institute of Food and Health, School of Public Health Physiotherapy and Sports Science, University College Dublin, Dublin, Ireland; ^3^Centre of Excellence for Public Health, Queen's University Belfast, Belfast, United Kingdom; ^4^UCD Institute of Food and Health, School of Public Health Physiotherapy and Sports Science, University College Dublin, Dublin, Ireland; ^5^School of Medicine, University College Dublin, Dublin, Ireland

**Keywords:** GWAS, immuno-metabolism, coronary artery disease, nutrition, omnigenic

## Abstract

Finding genetic variants that cause functional disruption or regulatory change among the many implicated GWAs variants remains a key challenge to translating the findings from GWAs to therapeutic treatments. Defining the causal mechanisms behind the variants require functional screening experiments that can be complex and costly. Prioritizing variants for functional characterization using techniques that capture important functional and regulatory elements can assist this. The genetic architecture of complex traits such as cardiovascular disease and type II diabetes comprise an enormously large number of variants of small effect contributing to heritability and spread throughout the genome. This makes it difficult to distinguish which variants or core genes are most relevant for prioritization and how they contribute to the regulatory networks that become dysregulated leading to disease. Despite these challenges, recent GWAs for CAD prioritized genes associated with lipid metabolism, coagulation and adhesion along with novel signals related to innate immunity, adipose tissue and, vascular function as important core drivers of risk. We focus on three examples of novel signals associated with CAD which affect risk through missense or UTR mutations indicating their potential for therapeutic modification. These variants play roles in adipose tissue function vascular function and innate immunity which form the cornerstones of immuno-metabolism. In addition we have explored the putative, but potentially important interactions between the environment, specifically food and nutrition, with respect to key processes.

## Introduction

CVD is increasing and the distribution of risk factors is changing with increasing prevalence of obesity and type II diabetes (T2D), particularly among young adults (aged 18–45) in developed countries (1–3). The burden of CVD risk factors remains very high because of unhealthy contemporary lifestyles, with dysregulated balance between energy intake and physical activity. In addition, malnutrition, wherein excess energy is coupled by micronutrient deficiencies, amplifies genetic risk (4). The major cardiovascular consequences of obesity and T2D predominantly derive from dysregulated and inflamed adipose tissue, particularly perivascular or visceral fat surrounding the organs (5). Visceral fat has limited expandability and becomes inflamed, with the resulting adipokine dysregulation adversely affecting vascular biology by promoting vasoconstriction, medial smooth muscle cell proliferation and endothelial dysfunction and is known as dysregulated immunometabolism (6). In the obese state, immune cells become activated and infiltrate metabolic tissues, chronic activation of inflammatory pathways in both vascular and immune components trigger stress kinase activation that impinge on the signaling of metabolic hormones such as insulin leading to impaired glucose and lipid homeostasis (7). Highly structured interactions between immune and metabolic responses are evolutionarily conserved and disruption of these interactions underlie many pathologies such as obesity and diabetes. Therapeutic solutions to tackle obesity, T2D and hypertension are drastically needed to reduce the overall burden of cardiovascular disease. However, many drugs or interventions have failed due to a lack of understanding of complex disease architecture (8, 9).

GWAs provided unique insights into the genetic architecture of complex diseases. Genetic architecture considers the overall composition of variants influencing a trait in terms of number, frequency and magnitude of effect and potential interactions, and can vary over traits (10). With increasing size and scope of GWAs, it has become clear that many complex traits are driven by enormously large numbers of variants of small effects. These variants are spread across the genome rather than in disease related pathways, include many without obvious connection to disease and/or related risk factors. These variants are potentially capturing most regulatory variants that are active in disease relevant tissues and the regulatory networks they form, may be so interconnected they affect the functions of core disease-related genes. This can be observed for variants that are heavily concentrated in regions that are transcribed or marked by active chromatin in disease-relevant tissues but with little enrichment for cell-type specific regulatory elements compared to broadly active regions. Boyle et al. (11) proposed that this pattern could be explained through an Omnigenic model of inheritance. This is an extension of RA Fischer's infinitesimal model of inheritance proposed nearly a century ago (12). The Omnigenic model considers that gene regulatory networks are so interconnected that all genes expressed in disease relevant cells are able to affect the functions of core disease-related genes. Most heritability is accounted for by effects of genes in peripheral pathways, outside of core pathways, which accounts for loci associated with multiple traits (pleiotrophy). Therefore, disease risk may be largely driven by genes with no direct relevance to disease and is propagated through regulatory networks to a much smaller number of core genes with direct effects.

In theory, the set of core genes must have a more pronounced effect on disease traits and proteins derived from these genes will drive pharmaceutical development and therapeutic strategies. However, how this works at the cellular regulatory network is incompletely understood. To understand the relevance of the variants for therapeutic development, it is crucial to understand their effect on protein level, activity or function. Even if a variant has a small effect on protein level and disease risk this protein may still be a suitable target for disease prevention if considered in context with its disease architecture (10). For instance, the SNP associated with (HMG-CoA reductase) explains 0.26% of variance in LDL levels, manipulating this gene can reduce LDL levels by 30–40% and reduce CAD risk (13). Even if the variants affect protein level or function, there are numerous challenges to drug development.

## An omnigenic architecture for cardiovascular disease?

GWAs has reproducibly associated over 160 variants with cardiovascular disease (14–20). By combining data from UK Biobank (34,541 cases and 261,984 non-cases) followed by replication in CardiogramplusC4D (88,192 cases and 162,544 controls), an additional 64 novel loci were recently prioritized (20). This identified a total of 163 loci associated with coronary artery disease (CAD) (21). Consistent with the omnigenic model for CAD genetic architecture, many novel candidate genes did not have an obvious connection to CAD and the genetic contribution was concentrated in regions transcribed or marked by active chromatin in relevant tissues (blood vessels and liver) but with little enrichment for cell-type specific regulatory elements. While they reconstituted a larger number of gene pathways/networks for CAD, increasing from 4 to 14%, overall the variants were spread throughout the genome and only 14% forming disease relevant pathways.

## Prioritizing variants using information from functional and regulatory regions

**Figure 1:**
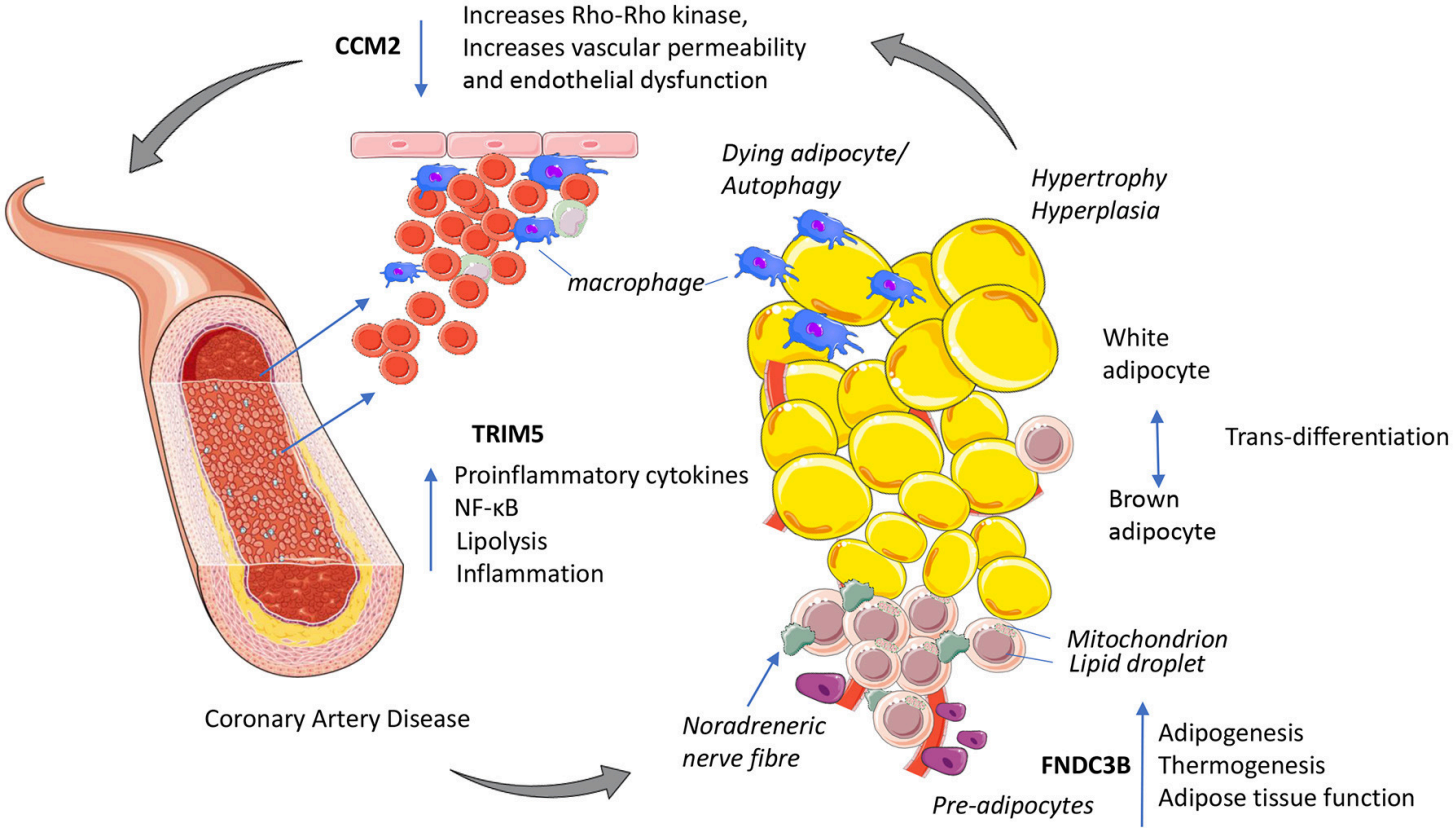
Putative mechanisms for three novel GWAs signals with functional links to immuno-metabolism and coronary artery disease. TRIM5 released from activated macrophages could increase proinflammatory cytokines NF-κB and shifts cellular energy from oxidative phosphorylation to lipolysis. CCM2 maintains endothelial function, decreased CCM2 increases Rho_Rho kinase activity increasing vascular permeability increasing inflammation. FNDC3B potentially enhances adipose tissue function by increasing adipogenesis and improving cellular energy efficiency by promoting oxidative phosphorylation and thermogenesis. This figure was prepared using the Servier medical art website (www.servier.fr).

To prioritize core CAD-related genes, they fine mapped the regions characterizing the functional, cellular and regulatory contribution of the variants (22) and prioritized their significance using probabilistic models (23) to derive a set of genes with converging evidence of potential functional SNP-gene mechanisms for functional follow up studies. The fine mapping methods that they used are compared against related methods summarized in Table 1, reviewed in Schaid et al. (30). Integrating information from multiple omics approaches in this way provides a more comprehensive understanding of the flow of information from the disease driver to its functional consequence or interactions. Methods can now test the mediating mechanism of these genetic variants on complex traits (31). Their analysis prioritized 161 variants to candidate genes based on proximity, expression quantitative loci data, DEPICT analysis and long-range chromatin interactions of variants with gene promotors for signals of regulation using stringent conditions and identified 28 loci with convincing arguments for causal variation, 22 known and 6 novel or 19 potential core genes (with missense, intergenic, downstream or UTR mutations). Among known genes; APOE, PCSK9, ANGPTL4 and SORT1 are implicated as core genes in lipid metabolism (a key component of immunometabolism) and targeting the effects of these genes can reduce CAD risk (32–34). Of the 6 novel signals, 3 are intergenic while 3 affect change through missense mutation or occur in a UTR3 region; these are TRIM5, FNDC3B, and CCM2 which are implicated in innate immunity, adipogenesis and vascular function, respectively, and all require functional follow up (Figure 1). Their study aimed to prioritize the CAD associations and elucidate regulatory connections that may influence the mechanism behind the associations, but according to the omnigenic model, broader regulatory connections between core genes must exist but are difficult to elucidate.

**Table 1:** Summary of methods for fine mapping variants from GWAs.

**Name**	**Combines**	**GWAs Data**	**Details**	**References**
BIMBAM, GUESS	GWA and phenotype	Individual level data	Stepwise conditional analysis on SNPs with lowest *p*-value association until no additional SNPs reach preassigned threshold for strength of association with phenotypic trait	(24, 25)
FINEMAP	GWA and phenotype	Summary level data	Use GWAs summary statistics and SNP correlations to compute Bayes factors for strength of association with trait. Uses a shotgun stocastic search which allows more variants to be considered simultaneously	(26)
CAVIARBF	GWA and phenotype	Summary level data	CAVIAR differs from PAINTOR by modeling the uncertainty in the observed association statistics. CAVIARBF has been reported to be more accurate than PAINTOR in prioritizing variants when no annotation information is available	(27)
GARFIELD (GWAS analysis of regulatory and functional information enrichment with LD correction)	GWAs, functional annotation and phenotype	Summary or individual level data	Select SNPs from LD blocks to prioritize variants matched with regulatory/functional annotation (of 1,005 specific regions selected from ENCODE, GENCODE and Roadmap Epigenetics) incorporating genic annotation, chromatin sites, histone modifications, DNAse I hypersensitivity sites, transcription factor binding sites from cell lines from ENCODE with their strength of association with traits	(22)
PAINTOR (Probability Annotation INTegratOR); fastPAINTOR	GWAs, functional annotation and phenotype(s)	Summary or individual level data	Selects SNPs from LD blocks allowing for multiple causal variants and matched with functional/regulatory annotation data (ENCODE), PAINTOR up-weights variants in certain functional annotations (e.g., transcription start sites) while downweighting variants within annotations less relevant to the trait (e.g., intergenic) without making *ad-hoc* assumptions on which tissue-specific annotations are relevant to the trait of interest. fastPAINTOR updates previous method leveraging pleiotrophy across correlated traits with a new sampling scheme to improve efficiency, it integrates fine mapping across two (multiple) traits assuming same variants impact both traits though allowing potentially distinct effect sizes/opposite effects.	(23, 28)
SMR (summary data based Mendelian randomization) and HEIDI (heterogeneity in dependent instruments)	GWAS, eQTL, mQTLs	Summary or individual level data	Combines summary level multi-omics data to prioritize gene targets and their regulatory elements in 3 steps, using association tests, 1. map methylome QTL to genes (2 MB), map expression QTLs to trait, map trait to mQTL, if signals significant in all 3 steps infers target genes functionalyl relevant, can incorporate info from two independent studies.	(29)

Several major challenges stand in the way to understanding how GWAs associations could become therapeutic targets. Most GWAs associations lie within non-coding regions making it difficult to predict their functions and identify targets/genes. Loci can be linked to multiple genes and the likely causal variant requires detailed investigation to elucidate the underlying mechanism. Functional follow up of important GWAs candidate loci now shows that multiple variants of small effect can synergistically drive dysfunction in regulatory networks, for example risk related to FTO (35), ANGPTL4 (17), GUCY1A3 (36), and SHROOM3 (37). To understand the mechanistic basis of increased adiposity associated with FTO, layers of OMICS data connecting epigenetic, gene co-expression and regulator expression followed by validation with genome editing elucidated the risk variant rs1421085 causes a loss of repression in AR1D5B which enhances expression of IRX3 and IRX5 increasing fat storage (35). Mining available OMIC data to gain insights into the complex regulatory circuitry behind these association signals has the potential to speed up functional follow-up by identifying novel links. We consider the three novel signals highlighted by van der Harst and Verweij for their strength of evidence and their importance to these pathways contributing to CAD risk or related traits such as adiposity and how these signals fit with other evidence supporting their contribution to disease risk. These may represent core genes but they may be signals that are context or cell specific to CAD. We also consider what the cell or tissue derived signals could offer therapeutically if they validated in independent studies. To this end, we explore a few examples wherein this paradigm may be relevant.

## Trim5, innate immune signaling and cad risk

The variant rs11601507 causes a missense mutation in TRIM5 and increases CAD risk (*p* = 2.1 × 10^−12^, OR 1.09 (95% C.I. 1.06, 1.11). Chromatin interactions between this variant and eQTLs in the promotors/enhancers of three other genes (TRIM6, OR52S1, OR52B6) suggest these genes enhance the expression of TRIM5. Chromatin interactions reveal relationships of chromatin organization in 3D space that may indicate biological function such as promotor-enhancer interactions. The evidence used to support rs11601507 is from a range of Hi-C experimental cell lines (20) (38). rs11601507 is a cis QTL for HBG2 (Hemoglobin) in whole blood (39) and shows significant tissue specific enrichment in veins and blood vessels [DEPICT analysis, (20)]. Ingenuity® pathway analysis (IPA®) prioritized TRIM5 and TRIM6 along with 14 other genes for association with CVD. IPA® considers upstream and downstream regulators of gene expression based on large scale causal networks (40). Interestingly, this same missense variant rs11601507 and a 5′UTR variant rs3824949 in TRIM5 has previously been associated with mean platelet volume (*p* = 6 × 10^−19^ and *p* = 1 × 10^−24^, respectively) (41) which is an example of pleiotrophy.

Given the enormous dimensionality of the phenome, it is unlikely that functional variants exist without pleiotrophic effects (42). Pleiotrophy can involve variants having effects on two or more traits via independent pathways (e.g., effects in different tissues) or effect of the variant in one trait causally related to variation in another trait. The risk allele of this variant has the same direction of effect for CAD and mean platelet volume. Using rs11601507 and other variants in a risk score, Astle et al. demonstrated a weak causal relationship between mean platelet volume and CAD risk using Mendelian randomization. Mean platelet volume is associated with increased hemolysis or free hemoglobin in the blood which is linked to increased inflammation. The TRIM5 association may be affecting both traits through inflammatory pathways.

TRIM5 promotes Interferon γ (IFNG) in macrophages, this forms part of the innate immune system (43). It has a capsid specific restriction factor that prevents infection from non-host adapted retroviruses. Interestingly, TRIM5 reciprocally enhances ubiquitination leading to co-operative action of IFNG and NF-κB pathways (44). There is a dynamic relationship between the innate immune system and metabolism, where re-configuration of energy metabolism between oxidative phosphorylation vs. glycolysis can define the immune-phenotype (45, 46). Fatty acids and other metabolites can influence and define immune cell functionality and cause metabolic reprogramming (45). It is hypothesized that this dynamic and reciprocal regulatory relationship between metabolism and inflammation plays a key role in metabolic disease including CAD (7). Macrophages play a key role as innate immune cellular mediators of inflammation. Activated macrophages can recruit other monocytes/macrophages to a developing lesion and increase lipid uptake and instigate metabolic stress and reprogramming in adipose tissue. Macrophages can become “metabolically activated” in the presence of glucose, insulin and palmitate. Metabolically activated macrophages demonstrate similar effects to classically activated macrophages, where both types activate the TLR4 and NF-κB pathways to promote pro-inflammatory cytokine secretion. However, the metabolically active macrophages also activate PPARγ, therefore controlling inflammation by prompting lipid metabolism (47). TRIM5 promotes IFNG and through a mechanism of decreasing tryptophan metabolism (which viruses rely on), IFNG inhibits the central metabolic regulator mTOR and metabolically reprograms macrophages to switch from glycolysis to oxidative phosphorylation and upregulates inflammation. CVD is associated with changes in many immune cell types at multiple sites of critical metabolic function with a cumulative detrimental effect on cholesterol, lipid and glucose homeostasis (7, 22).

## Olfactory signaling influences trim5 and is also linked to adiposity

The genetic mechanism associated with the TRIM5 variant suggests enhanced olfactory signaling enhances TRIM5 (innate immune signaling) to reduce lipolysis which enhances adiposity and increases risk of CAD. OR52B6, and OR52S1 are G-protein coupled olfactory signaling receptors (ORs) (48). These receptors interact with odorant molecules in the nose, to initiate a neuronal response that triggers the perception of a smell. OR52B6/OR52S1 have not been linked by GWAs signals as important regulatory variants but other variants related to olfaction have been have been significantly linked to obesity development through GWAS (8). Olfactory signaling is highly complex and can play a bidirectional role in controlling energy homeostasis in response to sensory and hormonal signals from the central nervous system (CNS) (49). Essentially the ORs may alter the drive to eat a poor diet, leading to obesity, hence elaborating an environmental insult. Reduced olfactory signaling increases β-adrenergic receptors on white (WAT) and brown adipose tissue (BAT) increasing lipolysis and fatty acid oxidation reducing obesity in mice (49). Olfaction influences the loss of function mutation in ADCY3 (50) gene and its interaction with the major obesity gene MC4R which disrupts ciliary targeting in neuronal cells critical for body weight regulation (35, 51). Heterozygous or homozygous null mice for ADCY3 are unable to smell (35).

In summary, innate immunity is important in the pathogenesis of CVD, here the association between a variant linked to innate immunity is reinforced and mediated through a novel mechanism of olfaction. The immunosuppressant drug cyclosporine is an antagonist for TRIM5 suggesting a potential therapeutic intervention is available to explore for functional relevance (20). More generally, targeting systemic inflammation through interleukin 1β (e.g., Canakinumab) has been shown to reduce CVD risk and by doing so has validated the inflammatory hypothesis of atherothrombosis (52). The variants TRIM5 and PROCR (*p* = 6.8 × 10^−12^) reaching GWAs significance are related to inflammation, which are relatively newly identified, show convergence between biological and genetic determinants of CVD and add to this inflammatory hypothesis (18, 20). An alternative therapeutic paradigm to anti-inflammatory modalities may be efforts to mimic the resolution of inflammation using specialized lipid mediators and their targets (53–55).

## FNDC3b, adipogenesis and cad risk

rs12897 is a common variant (MAF 0.41) showing a protective association with CAD; OR 0.96 (95% C.I. 0.95, 0.97) (*p* = 1.9 × 10^−10^), this SNP is an eQTL for the protein coding gene Fibronectin type III domain containing 3B (FNDC3B) (39) occurring in the 3′ UTR region of the mRNA likely affecting post-transcriptional regulation of gene expression. This SNP was the 3rd top gene prioritized by DEPICT (*p* = 1 × 10^−21^) in the overall analysis (20). IPA® prioritized a functional association between the protein of FNDC3B, TRIM5, TRIM6, VEGFA, and 12 other genes for association with CAD supporting a broader connectivity among these.

Adipogenesis is a key regulatory process, which determines adipose functionality, and its dysfunction is associated with metabolic-inflammation, hypoxia and related risks including insulin resistance (6) and deregulated cholesterol homeostasis and lipid metabolism (56) all of which lead to greater T2D and CVD risk (57). FNDC3B (alias FAD104) is a positive regulator of adipogenesis (58); specifically at the early stages of adipogenesis (59, 60). FNDC3B variant rs12897 was previously associated with large scale GWAs on height (*p* = 3 × 10^−39^) (61), waist-to-hip ratio (WHR) adjusted BMI (*p* = 8 × 10^−10^) and HIP adjusted BMI (*p* = 3 × 10^−12^) (62) and heart rate (*p* = 1 × 10^−9^) (63). Interestingly, intronic variants near FNDC3B strongly associated with intra-ocular pressure *p* = 9 × 10^−48^ (64) and *p* = 5 × 10^−50^ (65), however these variants are not in LD with rs12897. Although intraocular pressure may reflect changes in heart rate, this association may operate through a different, peripheral CAD pathway.

GWAs on specific adiposity traits and fat distributions (pericardial fat, visceral fat, WHRadjBMI, body fat percentage) have shown distinct genetic components (66, 67). WHR adjusted BMI and body fat percentage traits identified adipogenesis candidate genes to play key roles in adiposity. These genes included BMP2 (*p* = 3.3 × 10^−14^), CEBPA, PPARγ, HOXC-mir196, TBX15, and PEMT but these variants had no apparent regulatory links/eQTLs (62). While CEBPA and PPARγ are essential for white adipose tissue differentiation and are master regulators of adipogenesis, BMP2, like FNDC3B, is involved in early stage adipogenesis. FNDC3B and BMP2 are both involved in the early stage commitment of pre-adipocytes to proliferate and differentiate. FNDC3B (and BMP2) specifically induce and/or regulate the differentiation of committed progenitor cells toward adipogenesis or osteogenesis (68). Adipocytes and in particular pre-adipocytes are now recognized as more than fat-storing organelles having the capability to secrete cytokines and adipokines thus contributing to inflammation (69).

## Adipogenesis as a therapeutic mechanism to reduce metabolic risk

Defining the effectors which control the fate of adipocytes is of great interest to the therapeutic treatment of obesity. Obese individuals have a smaller proportion of brown adipose tissue (BAT) compared to white adipose tissue (WAT) which expands in response to lipid excess by hypertrophy, hyperplasia and inflammation and upon reaching a certain size become dysfunctional and necrotic, promoting macrophage infiltration. The conversion of WAT to the more functional energy dispersing BAT adipocytes would be a valuable approach to the treatment of obesity and its metabolic complications and is becoming the focus of anti-obesity research (9). Conversion of WAT to BAT can occur by two processes; adipogenesis (i.e., *de-novo*-adipocyte differentiation of precursor cells which FNDC3B may play a role) or more commonly trans-differentiation (i.e., WAT to beige/brite transition through molecular reprogramming, increasing mitochondrial oxidative phosphorylation/lipolysis requiring increased levels of uncoupling protein 1 (UCP-1) and enervated with b-adrenoreceptors (9). BAT derived from adipogenesis is more sensitive to stimuli from BMP7 (70) and BMP4 (71), irisin/FNDC5B (72), FGF21, and others. Irisin/FNDC5B is a myokine/cytokine that induces thermogenesis except in the obese state where it has a complex adaptive response to counterbalance decreased insulin sensitivity and other metabolic disorders associated with obesity (73) and is a key molecular target to induce browning of WAT (9). FNCD5B expression is highest at early stages of preadipocyte differentiation sharing 53% homology with FNDC3B but their relationship is unclear. While the eQTL affects the expression of FNDC3B, it is not known if this regulation is specific to a CVD relevant tissue or cell type. If regulation of FNDC3B is a key step in increasing adipogenesis, modulation of this process could enhance thermogenesis.

The strongest obesity variant associated to date, FTO, can act through a complex regulatory network also affecting pre-adipocyte differentiation highlighting the importance of this pathway (35). Interestingly, this regulation ensures it is restricted in a cell/tissue specific way to preadipocytes and mesenchymal adipocyte progenitors, not in brain or 120 other cell types (35). The causal variant associated with FTO disrupts AR1D5B binding in the risk haplotype leading to a loss of repression, this derepresses pre-adipocyte enhancer activity and increases IRX3 and IRX5 expression which represses mitochondrial thermogenesis and adipocyte browning making cells more likely to store fat.

## Other variants of genes influencing transdifferentiation of wat to bat are also linked to cad risk

In addition to FNDC3B, two other variants *PRDM16 and TWIST1* recently associated with CAD risk play key roles in adipogenic transdifferentiation of WAT to BAT which highlights this pathway as relevant to disease risk and therapeutic exploration. From a biological perspective PRDM16 is one of the most effective molecular targets to induce white-to-brown adipocyte trans-differentiation (9) and an intronic variant rs2493298 close to PRDM16 was recently identified to increase CAD risk (20). The variant rs2493298 *p* = 1.9 × 10^−9^, near PRDM16 occurs in an intronic region which physically interacts (chromatin, Hi-C experiments) with the promotors of three genes that act as enhancers (20) which have roles in metabolism (9). PRDM16 is essential for normal BAT function, it interacts with C/EBPβ and these are considered master regulators of BAT function (74), and functions in a feedback loop with PPARy and SIRT1. No pharmacological targets for PRDM16 are advanced enough to explore in clinical trials (9). PPARy coactivator 1 alpha (PGC1a) is another important control point of the BAT phenotype and it is repressed by TWIST1 which blocks target genes associated with PGC1a activity leading to browning of WAT (75). An eQTL rs21079595 intergenic to TWIST1 increases risk of CAD 1.3 × 10^−24^ and was prioritized as a core CAD related gene (20). Previously this variant had been linked to HDAC9 gene through proximity, but expression data from the Stockholm-Tartu Atherosclerosis Reverse Network Engineering Task Study (STARNET) in two different tissues prioritized this eQTL variant to TWIST1 (76). Manipulation of PGC1a can also achieve reductions in inflammatory disease risk and enhance adipogenesis through dietary fat modification (6).

In summary, several variants associated with adipogenesis have been associated with CAD risk. Dysregulation of adipocyte browning/thermogenesis, particularly in visceral fat surrounding thoracic and aortic arch, is important in the pathogenesis of CVD. FNDC3B is among several variants that impact adipogenesis, while the regulatory networks among these still need more complete understanding, manipulation of this pathway at the preadipocyte stage could impact CVD risk.

## CCM2, endothelial function and CVD risk

The variant rs2107732 causes a missense mutation in the CCM2 gene and is associated with reduced risk of CAD (OR 0.94 (95% C.I. 0.93–0.96), *p* = 3.6 × 10^−8^ (20). A variant in the promotor of MYOG1 forms a chromatin interaction with the CCM2 variant suggesting this regulates CCM2 (20). MYOG1 is a muscle specific transcription factor that induces myogenesis (muscle formation). Mutations in an orthologous mouse gene of CCM2 cause a cardiovascular phenotype in mice and mutations in MYOG1 caused abnormalities in inflammation/white blood cells of mice (Mouse Genome Informatics database) (20). Inherited loss-of-function mutations in CCM2 (and also CCM1 and CCM3 genes) are implicated in abnormal vascular morphogenesis and can cause vascular lesions called cerebral cavernous malformations which develop in the human CNS (77). CCM2 is expressed in the brain and heart and CCM genes (including CCM2, CCM1, CCM3) are crucial regulators of heart and vessel formation and integrity by restricting vascular permeability and maintaining vascular homeostasis (78–80). These genes form complexes but also have complex independent roles (81). CCM2 restricts vascular permeability and maintain endothelial barrier function (tight and adherens junctions) by inhibiting Rho A-Rho kinase activity by enhancing Rho A proteasome degradation (79, 82). A lack of CCM2 increases Rho A Rho kinase activity which disrupts endothelial cell-cell contact causing permeability and stress fiber formation„ which is the initial phase in many cardiovascular diseases and characteristic of pathologically activated vascular endothelium. The response of CCM2 may be different in the inflammatory state and the MYOG1 transcription factor may influence this under certain conditions. Endothelial dysfunction reduces the ability of arteries to fully dilate, which stimulates vasodilators from the endothelium like nitric oxide (NO), decreased availability of NO or inactivation due to reactive oxygen species increases dysfunction. An intronic variant in NOS3 is also prioritized as a core causal variant of CAD alongside CCM2 both being important to blood vessel morphology and function (20) with several other rare and common variants in GUCY1A3 PDE5A and PEDE3A (16, 20, 36, 83, 84) highlighting the importance of the NO/cGMP signaling pathway to atherosclerosis and CAD risk.

Increased vascular permeability correlates with neo-angiogenesis (80). CCM proteins also control angiogenesis via Rho-kinase and other signaling pathways (78), CCM2 inhibits angiogenesis, loss of CCM2 causes dramatic angiogenic remodeling abnormalities (85). Adipose tissue is probably the most highly vascularized tissue in the body, as each adipocyte is encircled by capillaries, angiogenesis plays a key role in its function (86). Angiogenesis is driven by a complex interplay of angiogenic factors and inhibitors including vascular endothelial growth factor A (VEGFA). VEGFA is among the top 64 novel CAD loci increasing risk of CAD (OR 1.95 (95% C.I. 1.03–1.06), *p* = 1.9 × 10^−12^) (20) and waist to hip ratio adjusted BMI *p* = 3 × 10^−27^ (62). CCM proteins and particularly CCM3 can regulate VEGFA expression (80) [typically CCM2 and CCM3 function as a complex, (81)]. Lipid accumulation in adipocytes activates Rho Rho kinase signaling by breaking endothelial cell barriers/stress fiber formation triggering inflammatory changes (87). Vascular remodeling determines the flexibility and metabolic rate of adipose tissue and the communication between adipose and endothelial cells is crucial. Dysfunctional communication in obese individuals contributes to development and progression of T2D including impaired vasodilation, hypoxia and inflammation. CCM2 and VEGFA play roles at the interface of this cellular communication. A more complete understanding of the regulatory networks connecting CCM2 (inhibits angiogenesis) and VEGFA (stimulates angiogenesis, of which there are already targeted drugs), might synergistically increase the resulting therapeutic efficacy to combat obesity and CVD.

## GWAs targets with functional links to immuno-metabolism and coronary artery disease

In summary, the three novel GWAs signals implicated in CAD risk play putative roles in immuno-metabolism (Figure 1). TRIM5 has potential to increase innate immunity, inflammation and CAD risk via macrophage infiltration of adipose tissue increasing metabolic stress. Activation of pro-inflammatory immune cells requires a shift to move from energy efficient oxidative phosphorylation to anaerobic glycolysis favoring glucose as substrate. This break or shift occurs when macrophages become polarized (M1) and is associated with nitric oxide production; an M1 effector molecule triggered by increasing oxidative stress. The mutation in CCM2 may reduce oxidative stress to maintain endothelial function, control angiogenesis and vascular remodeling of blood vessels including those surrounding adipose tissue to reduce CAD risk. Inhibiting glycolysis promotes the resolution of inflammation. FNDC3B enhances adipose tissue function by increasing adipogenesis and improving cellular energy efficiency by promoting oxidative phosphorylation and thermogenesis, with PRDM16 and TWIST1 playing similar roles in modifying CAD risk (Figure 1).

## Dietary interventions connecting adipogenesis and metabolic inflammation as therapeutic mechanisms to reduce metabolic risk

Since the recognition that fatty acids can modulate an inflammatory response, e.g., via lipid induced re-programming of macrophage metabolism and inflammation (88) or the NLRP3 inflammasome (89, 90) they have been studied for their immunomodulatory effect on insulin resistance and dysregulated lipid metabolism pathways. Dietary manipulation and certain nutrients have the potential to modulate inflammatory responses. Obesity promotes adipose hypertrophy, with inflammation interacting with the adipogenic process. Pro-inflammatory cytokines IFNG, IL-1β and TNFα, inhibit adipogenesis by downregulating PPARy and C/EBP (91–94) and several dietary components can modulate this effect e.g., reservatrol, flavonoids and polyphenols (95–97). Dietary fat modification to replace saturated fatty acids (SFA) with monounsaturated fatty acids (MUFA) and polyunsaturated fatty acids (PUFA) may provide a potential strategy to lessen inflammation that enhances adipogenesis to attenuate insulin resistance and dysregulated lipid metabolism (6) but the impact of dietary fat modification in humans has varied (98).

Efforts to explain this inter individual variability in response, has focused on the interaction between the genes, metabolites and diet. As diet is the exogenous source of many metabolites, as well as affecting the generation of endogenous metabolites, interactions with the nutritional environment are plausible. However, many putative gene/variant-diet associations have failed to replicate in large studies (99) with various approaches to enhance power (100). Some intriguing examples of specific variant-metabolite interactions modulating disease risk exist from small studies (101, 102). The variant, rs5082 of APOA2 interacts with SFA intake to influence risk of obesity (101). This is modulated through an epigenetic effect on APOA2 regulatory region which promoted an APOA2 expression difference between APOA2 genotypes on a high SFA diet. This selectively dysregulated branched chain and tryptophan metabolic pathways with possible implications for food intake.

## Understanding the regulatory networks underlying metabolic traits

Attention is shifting to large scale studies integrating transcriptomic and metabolomic data to understand the interplay between genes and metabolites (103). To explore genes playing key roles in immunometabolism more specifically, Nath et al. integrated transcriptomics (focusing on immune networks) and metabolomics using 2,168 individuals from two general population cohorts (104). They identified significant expression quantitative loci in 8 immune gene networks highlighting the genetic foundations of these effects. For example, an eQTL in the ARHGEF3 gene (rs1354034 *p* = 7 × 10^−28^) had trans regulatory effects on several genes associated with platelet function and this module had diverse effects on 55 metabolites. Other important core immunometabolic associations related to neutrophil activation and viral response. A subset of the cohort measured repeatedly over 7 years, demonstrated the gene-metabolite effects were temporally stable (104). As long-term OMICs data will be collected on population cohorts over time, these signals are likely to become more reliable.

Identifying the genetic basis to these interactions can be useful therapeutically to modulate the variant itself for individualized treatment or modulate the pathway the variant functions in, which can have much wider implications for population treatment e.g., PCSK9 inhibitors for individual and population level treatment of hypercholesterolemia and CVD risk (34). With better understanding of metabolite-immune interactions, *in vivo* and interventional studies can be developed to modulate these interactions through existing lipid lowering medications, gut microbe effects or dietary changes. In this way, the immune system itself can be harnessed to reduce the burden of cardiovascular and metabolic disorders (55, 71). With distinct lifestyle strategies now known to differentially affect the way adipose tissue is stored and utilized in the body (105), it is important to understand where and how the drivers of these regulatory networks are acting, which might be under specific situations or locations.

## Cellular specificity of regulatory networks

Progress has been made to determine the tissues and cell types underlying disease through the GTex consortium (106). GTex, Roadmap Epigenomics and Functional Annotation of Mammalian Genomes 5 (FANTOM5) provides reference sets for multi-tissue gene expression and epigenomics consistently evaluated on the same individuals with available tissues. Different layers of regulation can exist from post-transcriptional, post-translational, protein-protein interactions and intercellular signaling, mediated through chromatin interactions and expression quantitative trait loci. Assuming that most of the regulation occurs through genes (linked by eQTLs), regulation can occur at the tissue level, broad cell population level or in very specific cell types (106–110). For CVD, multiple cell types or highly specialized cell types may be involved (e.g., vascular, liver, adipose) where cellular networks could have variable expression across cell types (111). The effect of particular variants would then be an average of its effect size in each cell type weighted by cell type importance (11). Mapping GWAs signals to promotors/enhancers measured by cap analysis gene expression (CAGE) found regulation for specific diseases could be turned on/off in similar complex patterns across different cell types. For example, shared cell type specific regulatory networks distinguishing two sub-types of ulcerative colitis could be distinguished based on regulatory signals guided by GWAs signals enriched in either monocytes exposed to inflammatory signals or epithelial cells (108).

To identify cell type specific gene regulation, grouped cell types or deconvolution methods have been used, but the methods tend to be biased to specific cell types or difficult-to-identify less abundant cell types (112). It is possible to calculate the probability a GWAs variant and eQTL tag the same functional effect and infer the tissues where the effect for a trait is likely manifested (113). Single cell RNAseq (scRNAseq) can identify cell-type or context specific eQTLs, but the requirement for fresh tissue and costs limits large scale screening. Mapping monogenic kidney mutations or genome-wide variants associated with chronic kidney disease to gene expression from scRNAseq of 57,979 mouse kidney cells, Park et al. inferred that these variants were expressed in only one particular cell type (114). This suggests, most genetic diseases of the kidney can be traced to single cell types. Using intercellular variation from expression profiles from 25,000 peripheral blood mononuclear cells from 45 donors, scRNAseq identified cell type specific cis-eQTLs. Although gene regulatory networks were highly personal, their approach identified more genes under genetic control or specific cell type in which the effect is most prominent and found examples of SNPs influencing the co-expression of 2 genes (115). scRNAseq has the potential to group and examine the effects of cells along the cell cycle, along a differentiation path (e.g., adipocyte differentiation) or along a response to an environmental stimulus (e.g., inflammatory signaling) (116). With improved understanding of how these genes impact cell types and tissues, more specific targeted interventions can be developed, for instance improved drugs, mobilizing specific fat deposits (105) or nutritional interventions (117).

## Conclusions

We have highlighted three novel variants associated with CAD risk which have been prioritized and annotated based on systems genetics approaches including expression quantitative trait analysis and network analysis to infer their functional relevance. These core variants play roles in innate immunity, adipogenesis and endothelial function which drive coronary artery disease and principally in the role that obesity and T2D shape the pathogenesis of CAD through immuno-metabolism. Core variants representing these pathways provide a starting point to potential mechanism that may lead to therapeutic manipulation with further understanding of the regulatory networks connecting these is needed. Given that CAD is a multifactorial disease, it may be possible in the future to develop individual treatment strategies based on these variants or design relevant population level interventions based on the pathways these variants highlight for subsets of people in the population with subtypes of CAD risk related to obesity or T2D.

## Author contributions

MH wrote the paper. YL, CG, and HR discussed and wrote sections, refined and edited the text. All agree with the submission.

### Conflict of interest statement

The authors declare that the research was conducted in the absence of any commercial or financial relationships that could be construed as a potential conflict of interest.
